# Immunalysis tapentadol assay reformulation resolves tramadol interference

**DOI:** 10.1093/jat/bkaf060

**Published:** 2025-06-30

**Authors:** Adeolu O Adegoke, Alexandria N Jackson, Sonia L La’ulu, Chelsie Anderson, Joseph W Rudolf, Jessica M Boyd, Kamisha L Johnson-Davis

**Affiliations:** Department of Pathology, University of Utah Health, Salt Lake City, UT 84132, United States; ARUP Laboratories, Salt Lake City, UT 84108, United States; ARUP Institute for Clinical and Experimental Pathology, Salt Lake City, UT 84108, United States; ARUP Laboratories, Salt Lake City, UT 84108, United States; ARUP Institute for Clinical and Experimental Pathology, Salt Lake City, UT 84108, United States; ARUP Laboratories, Salt Lake City, UT 84108, United States; ARUP Laboratories, Salt Lake City, UT 84108, United States; Department of Pathology, University of Utah Health, Salt Lake City, UT 84132, United States; ARUP Laboratories, Salt Lake City, UT 84108, United States; Department of Pathology, University of Utah Health, Salt Lake City, UT 84132, United States; ARUP Laboratories, Salt Lake City, UT 84108, United States; Department of Pathology, University of Utah Health, Salt Lake City, UT 84132, United States; ARUP Laboratories, Salt Lake City, UT 84108, United States

## Abstract

This study evaluated the performance of the Immunalysis Tapentadol 343 Urine Enzyme Immunoassay (EIA) screening kit, focusing on the prevalence of false-positive results due to cross-reactivity with tramadol. Tapentadol is a dual-action analgesic, modulating μ-opioid receptors and inhibiting norepinephrine reuptake, while tramadol, a structurally related compound, is a weak μ-opioid receptor agonist and norepinephrine/serotonin reuptake inhibitor. Cross-reactivity between these compounds can complicate urine drug screening results for adherence monitoring in chronic pain management. A total of 28 samples initially produced false-positive results for tapentadol BNl using the Immunalysis Tapentadol 343 Urine EIA screening kit. Liquid chromatography–tandem mass spectrometry (LC–MS/MS) was used to confirm the absence of tapentadol. Of the false-positive samples, 61% contained tramadol at concentrations below the manufacturer-reported cross-reactivity threshold of 60 000 ng/mL, indicating assay limitations in specificity. To address this issue, a newly reformulated Immunalysis Tapentadol 343UR Urine HEIA kit was evaluated for tramadol cross-reactivity. Upon retesting the 28 false-positive samples with the reformulated kit, no false positives were detected, with results consistent with LC–MS/MS confirmation. The rate of false-positive tapentadol screens in urine has substantially reduced since the implementation of the new tapentadol kit in routine testing. These findings demonstrate the importance of assay verification to assess cross-reactivity, particularly for structurally related compounds. The reformulated Immunalysis Tapentadol 343UR kit shows improved specificity, reducing false-positive rates and enhancing the accuracy of tapentadol detection in clinical and forensic toxicology applications.

## Introduction

Chronic pain remains a prevalent and challenging condition, necessitating the development of effective analgesics with improved safety profiles. Tapentadol, introduced as a novel analgesic, is characterized by its dual mechanism of action, designed to address pain through μ-opioid receptor (MOR) modulation and norepinephrine (NE) reuptake inhibition [1, 2]. A similar analgesic, tramadol, demonstrates modest agonistic effects on MOR and concurrently acts as an inhibitor of serotonin and NE reuptake [3].

Tapentadol is listed under Schedule II of the United States Controlled Substances Act [4], and adherence/compliance monitoring to prescribed medication and/or misuse of tapentadol can be evaluated by urine drug screens (UDS), which may employ an immunoassay (IA) technique to identify the presence of the parent drug or its metabolite in urine. IAs are widely used in UDS due to their ease of use, automation adaptation, and quick turnaround time. However, analyte-specific antibody cross-reactivity with structurally related and unrelated compounds in sample matrices is a major limitation of IA, potentially resulting in false-positive outcomes [5, 6]. To minimize reporting false-positive UDS, confirmatory testing with gas chromatography–mass spectrometry (GC–MS) or liquid chromatography–tandem mass spectrometry (LC–MS/MS) is recommended [6].

The high costs of performing MS testing, longer turnaround time to results, and limited availability of MS methods leave the care providers with decisions regarding patient care based on “presumptive-positive” drug screening results until confirmatory testing is performed. While unconfirmed IA screening results should never be used to make definitive clinical or legal decisions, in practice, such results are sometimes inappropriately acted upon. This misuse can lead to serious consequences, including inappropriate removal from pain management programs, adverse employment actions, or unjustified involvement of child and family services [7, 8].

In addition, physicians may also have knowledge gaps regarding drug metabolism and assay limitations, such as IA cross-reactivity, the risk of false positive and false negative results, when interpreting UDS tests, which can negatively impact patient care [9–11].

In this study, we investigated the cause of increased false-positive tapentadol results in our UDS assay, utilizing both IA and LC–MS/MS methods. Our findings shed light on the complexities involved in accurately screening tapentadol in urine samples using IA method, particularly in the presence of high tramadol concentrations.

## Materials and methods

### Patient samples

Previously analyzed random urine samples were saved for analysis in compliance with the University of Utah Institutional Review Board (IRB #00082990). Specimens were stored at −20°C until analysis. After thawing, urine samples were mixed thoroughly before taking aliquots. The age range of the patients whose samples were selected was 34–85 years old, and their sex distribution was 9 males and 19 females.

### Synthetic urine preparation

Synthetic urine was prepared in nanopure water by ARUP Laboratories Reagent Lab, (Salt Lake City, UT, USA) with the following constituents, and final concentrations: 0.33 M urea, 0.12 M sodium chloride, 0.016 M potassium diphosphate, 0.007 M creatinine and 0.004 M sodium monophosphate.

### Immunoassay kits

UDS was performed using the Drug Profile, Screen with Reflex to Quantitation assay at ARUP Laboratories (Test Code: 2012312). The screening utilized IA reagents from multiple manufacturers, processed on the Beckman Coulter AU5800 Series Clinical Chemistry Analyzer. The following reagent kits were used: Tapentadol 343 Urine EIA (Old Kit-343, Cat. #343-0500) and Tapentadol 343UR Urine HEIA (New Kit-343UR, Cat. #343UR-0500) from Immunalysis Corporation, Pomona, CA, USA; Tramadol (Cat. #5040-0001-02) and Ethyl Glucuronide (EtG) from ARK Diagnostics, Inc., CA, USA; Emit^®^ II Plus Drug Screening for opiates, amphetamines, cannabinoids (THC), benzodiazepines, and methadone from Siemens Healthcare Diagnostics Inc., DE, USA. The screening cutoff concentration for each analyte is: tapentadol = 200 ng/mL, tramadol = 100 ng/mL, EtG = 500 ng/mL, opiates = 300 ng/mL, amphetamines = 300 ng/mL, THC = 50 ng/mL, benzodiazepines = 200 ng/mL, and methadone = 150 ng/mL.

### Mass spectrometry confirmation

All presumptive-positive UDS IA results were confirmed by LC–MS/MS at ARUP Laboratories [12–16]. Briefly, deuterated analogs of tramadol, *O*-desmethyltramadol, and tapentadol were used as internal standards (IS) and added to aliquots of urine specimens to account for variability during extraction and analysis. This method does not include a hydrolysis step. Tramadol and *O*-desmethyltramadol were extracted using solid-phase extraction, while tapentadol was separated using liquid protein crash extraction. The tramadol extracts were reconstituted in mobile phase and analyzed using an AB Sciex Triple Quad 5500 LC–MS/MS system with positive-mode electrospray ionization, and the tapentadol extracts were injected into an Agilent 6470 Triple Quad LC–MS/MS, both instruments are equipped with an Agilent 1260 Infinity II Rapid Resolution HPLC. Analytes were separated by HPLC, followed by fragmentation of selected precursor ions into product ions by collision with neutral gas molecules, with multiple reaction monitoring used to track the transitions. Data acquisition and analysis were performed using AB Sciex Analyst and Indigo^®^ software, with quantitation based on comparison to calibration curves. A specimen was reported as positive if the drug concentration exceeded the confirmation cutoff concentration (i.e. tapentadol = 50 ng/mL; tramadol and *O*-desmethyltramadol = 25 ng/mL). Calibrators and controls were processed alongside the specimens to ensure calibration and procedural verification.

### Statistical analysis

Statistical analysis and data visualization were performed in Microsoft Excel (Microsoft Corporation, Redmond, WA, USA) and GraphPad Prism (GraphPad Software, La Jolla, CA, USA).

## Results

**Figure 1.**
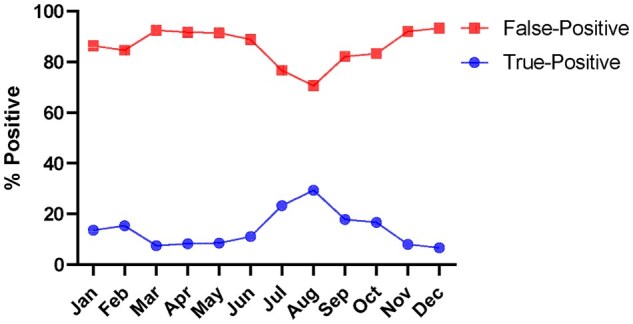
Percentage of false-positive tapentadol UDS samples among the screen positive cases between January and December 2023.

Presumptive-positive Tapentadol IA UDS were automatically subjected to confirmation by LC–MS/MS. However, persistently elevated rates of false-positives in the tapentadol IA were observed over 12 months. The percentage of false-positive tapentadol UDS calculated as [(# of samples Confirmed Negatives (CN)/# of samples that are Screen Positives (SP)) x100] was persistently >70% (Fig. 1).

**Figure 2.**
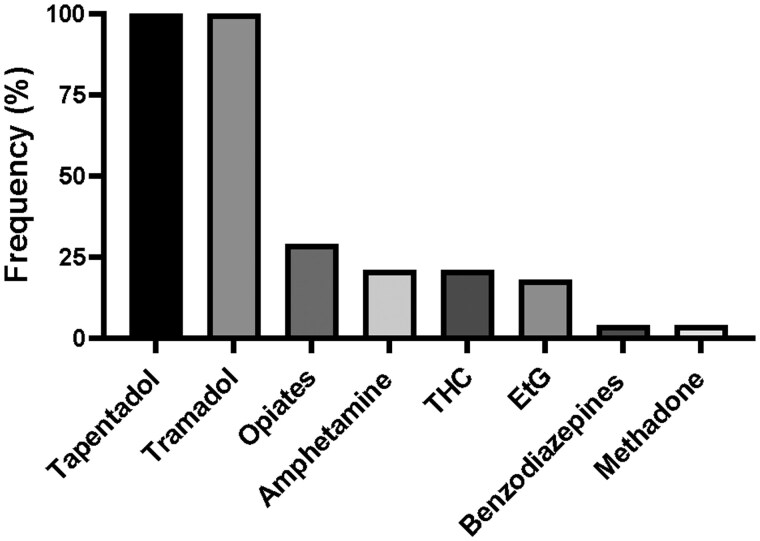
Percentage frequency of drug analyte positivity in the urine samples (*n* = 28) with false-positive tapentadol IA UDS.

To characterize the false-positive tapentadol UDS, 28 samples that screened presumptively-positive for tapentadol were selected for screening by our UDS panel, which also screens for opiates, methadone, benzodiazepine, tramadol, EtG, THC, and amphetamine. The drug screen results for these patients were evaluated, and all 28 (100%) samples, that were presumptive-positives for tapentadol, were also presumptively- positive for tramadol (Fig. 2). However, only 29% of the screened samples were presumptively-positive for opiates, 21% were presumptive-positives for amphetamine and THC, 18% were presumptively-positive for EtG, and 4% were presumptive-positives for benzodiazepines and methadone (Fig. 2). This suggests that tramadol might cross-react with the tapentadol IA to generate false-positive tapentadol results.

Per the package insert of Old Kit-343 (Revision: E.0, Revision Date: 09/2015), a tramadol concentration of 60 000 ng/mL, generated 0.3% cross-reactivity with tapentadol UDS (assay cutoff = 200 ng/mL). Although cross-reactivity of 0.3% is negligible, we speculated that the 28 samples with false-positive tapentadol UDS would have tramadol concentrations significantly higher than 60,000 ng/mL (i.e. the tramadol concentration where cross-reactivity was reported in the package insert). To examine this, quantitative LC–MS/MS analysis was performed, which showed the concentrations of tramadol and its metabolite, *O*-desmethyltramadol, in the 28 samples were between 6901 and 297 703 ng/mL and 1479–85 956 ng/mL, respectively (Table 1). Eleven out of 28 (39%) false-positive tapentadol samples had tramadol concentrations > 60 000 ng/mL, with 2 of these 11 samples (18%) having *O*-desmethyltramadol concentrations of > 60 000 ng/mL (Table 1). Meanwhile, all the false-positive samples had tapentadol concentrations of <50 ng/mL as confirmed by the LC–MS/MS method (Table 1).

**Table 1.** Tramadol LC–MS/MS data for samples with false-positive tapentadol screen by IA

ID	Tapentadol (ng/mL)	Tramadol (ng/mL)	*O*-desmethyltramadol (ng/mL)
E01	<50	30 000	8503
E02	<50	50 809	6810
E03	<50	**260 944**	30 174
E04	<50	52 389	3834
E05	<50	**70 675**	6494
E06	<50	53 063	14 020
E07	<50	37 702	14 795
E08	<50	**107 131**	54 062
E09	<50	13 908	5037
E10	<50	6901	6854
E11	<50	59 859	16 688
E12	<50	**183 353**	29 200
E13	<50	17 317	2797
E14	<50	14 788	11 040
E15	<50	**297 703**	**85 956**
E16	<50	17 180	6487
E17	<50	28 471	5943
E18	<50	20 833	6413
E19	<50	**94 524**	30 268
E20	<50	**78 893**	10 699
E21	<50	37 367	13 670
E22	<50	**64 781**	15 129
E23	<50	13 172	6174
E24	<50	**75 270**	21 447
E25	<50	10 027	1479
E26	<50	**94 475**	11 741
E27	<50	23 806	7795
E28	<50	**235 395**	**71 029**

Numbers in bold text denote samples with tramadol or *O*-desmethyltramadol concentrations > 60 000 ng/mL.

In contrast to tramadol cross-reactivity at 60 000 ng/mL in tapentadol Old Kit-343, the package insert of the New Kit-343UR states that tramadol concentration of 100 000 ng/mL, equivalent to 200 ng/mL of tapentadol (assay cutoff = 200 ng/mL), generated < 0.2% cross-reactivity with tapentadol UDS. To examine the performance of the New Kit-343UR versus Old Kit-343, we made three aliquots of low (150 ng/ml) and high (250 ng/ml) tapentadol controls. One aliquot each of the low and high tapentadol control was left unspiked (“Neat”), spiked with tramadol (Tram), and spiked with *O*‐desmethyl‐tramadol (*O*Tram). To replicate the tramadol cross-reactivity cutoff of Old Kit-343 (i.e. 60 000 ng/mL) and New Kit-343UR (i.e. 100 000 ng/mL), a slightly lower concentration (i.e. 48 000 ng/mL), and a slightly higher concentration of Tram and *O*Tram (i.e. 65 000 ng/mL and 107 000 ng/mL) were spiked into the low and high tapentadol controls (Table 2). As expected, UDS with the tapentadol Old Kit-343 showed a presumptive-negative tapentadol screen for the low tapentadol controls not spiked with tramadol (Tap-Lo-Neat) and a presumptive-positive tapentadol screen for the high tapentadol controls not spiked with tramadol (Tap-Hi-Neat). In contrast, a presumptive-positive tapentadol screen (100%) was obtained with the Old Kit-343 in the low tapentadol controls spiked with Tram or *O*Tram, regardless of their concentrations. In addition, the IA ARK Tramadol assay showed a presumptive-negative tramadol screen in the unspiked “Neat” controls, and a presumptive-positive screen in the Tram and *O*Tram spiked tapentadol controls (Table 2). These IA results were confirmed by quantitative LC–MS/MS method, which showed the concentration of each analyte in the synthetic urine (Table 2). Although the New Kit-343UR showed a superior performance in comparison to Old Kit-343, Tap-Lo-*O*Tram-01 and Tap-Lo-*O*Tram-02 were falsely positive with the New Kit-343UR, suggesting that *O*Tram is still cross-reacting at concentrations between 60 000 ng/mL—107 000 ng/mL (Table 2).

**Table 2.** Tramadol cross-reactivity in tapentadol UDS

Assay		Old Kit-343	ARK Tram	LC–MS/MS			New Kit-343UR
**Analyte**		**Tap**		**Tap**	**Tram**	***O*Tram**	**Tap**
**Cut-off (ng/mL)**		**200**	**100**	**50**	**25**	**25**	**200**
**ID**	**Spiked analytes (ng/mL)**	**Qual Result**	**Qual Result**	**Quant Result**	**Quant Result**	**Quant Result**	**Qual Result**
Tap-Lo-Neat-01	Tap (150)	Neg	Neg	172	0	0	Neg
Tap-Lo-Neat-02	Tap (150)	Neg	Neg	185	0	0	Neg
Tap-Lo-Neat-03	Tap (150)	Neg	Neg	134	0	0	Neg
Tap-Hi-Neat-01	Tap (250)	Pos	Neg	280	0	0	Pos
Tap-Hi-Neat-02	Tap (250)	Pos	Neg	265	0	0	Pos
Tap-Hi-Neat-03	Tap (250)	Pos	Neg	329	0	0	Pos
Tap-Lo-Tram-01	Tap (150) Tram (107 000)	Pos	Pos	156	119,943	0	Neg
Tap-Lo-Tram-02	Tap (150) Tram (65 000)	Pos	Pos	162	53,845	0	Neg
Tap-Lo-Tram-03	Tap (150) Tram (48 000)	Pos	Pos	162	40,430	0	Neg
Tap-Hi-Tram-01	Tap (250) Tram (107 000)	Pos	Pos	242	86,043	0	Pos
Tap-Hi-Tram-02	Tap (250) Tram (65 000)	Pos	Pos	267	54,480	0	Pos
Tap-Hi-Tram-03	Tap (250) Tram (48 000)	Pos	Pos	282	36,892	0	Pos
Tap-Lo-*O*Tram-01	Tap (150) *O*Tram (107 000)	Pos	Pos	147	0	136,811	Pos
Tap-Lo-*O*Tram-02	Tap (150) *O*Tram (65 000)	Pos	Pos	148	0	65,920	Pos
Tap-Lo-*O*Tram-03	Tap (150) *O*Tram (48 000)	Pos	Pos	151	0	46,638	Neg
Tap-Hi-*O*Tram-01	Tap (250) *O*Tram (107 000)	Pos	Pos	231	0	121,920	Pos
Tap-Hi-*O*Tram-02	Tap (250) *O*Tram (65 000)	Pos	Pos	246	0	53,400	Pos
Tap-Hi-*O*Tram-03	Tap (250) *O*Tram (48 000)	Pos	Pos	270	0	45,840	Pos

Abbreviations: Tap, tapentadol; Tram, tramadol; *O*Tram, *O*‐desmethyl‐tramadol; Qual, qualitative; Quant, quantitative; Neg, negative; Pos, positive.

**Figure 3.**
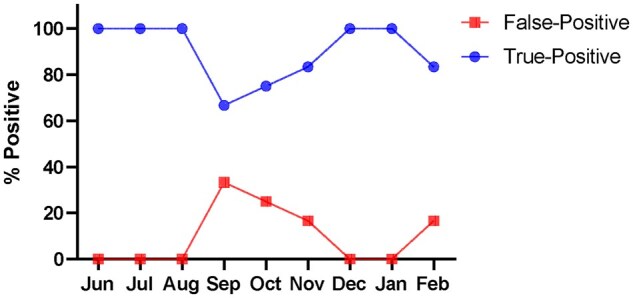
Percentage of false-positive tapentadol UDS samples among the screen positive cases between June 2024 and February 2025.

Having observed the improved performance of the New Kit-343UR, we re-tested the 28 false-positive patient samples in Fig. 2 and compared it with the Old Kit-343. In contrast to 100% false-positive results obtained with the Old Kit-343, all 28 (100%) samples were negative for tapentadol with the reformulated New Kit-343UR (Table 3), which was consistent with the results obtained with LC–MS/MS analysis in Table 1. This confirmed that the reformulated New Kit-343UR was less affected by tramadol cross-reactivity. Our lab switched to the new tapentadol reformulation kit in May 2024. Figure 3 shows a 34% reduction in the false positive rate with the New Kit-343UR, for June 2024—February 2025.

**Table 3.** Tapentadol UDS comparison using New Kit-343UR and Old Kit-343

Assay	New Kit-343UR		Old Kit-343	
Cut-off	200 ng/mL		200 ng/mL	
Cut-off Normalized	100 ng/mL		100 ng/mL	
ID	**Result** ^a^	Interpretation	**Result** ^a^	Interpretation
E01	20	Neg	112	Pos
E02	31	Neg	120	Pos
E03	55	Neg	160	Pos
E04	8	Neg	122	Pos
E05	15	Neg	117	Pos
E06	11	Neg	134	Pos
E07	21	Neg	121	Pos
E08	50	Neg	118	Pos
E09	26	Neg	132	Pos
E10	9	Neg	106	Pos
E11	23	Neg	156	Pos
E12	52	Neg	120	Pos
E13	12	Neg	126	Pos
E14	22	Neg	109	Pos
E15	86	Neg	150	Pos
E16	9	Neg	113	Pos
E17	23	Neg	155	Pos
E18	12	Neg	128	Pos
E19	33	Neg	111	Pos
E20	19	Neg	115	Pos
E21	21	Neg	107	Pos
E22	25	Neg	113	Pos
E23	6	Neg	100	Pos
E24	51	Neg	114	Pos
E25	12	Neg	109	Pos
E26	25	Neg	145	Pos
E27	28	Neg	139	Pos
E28	71	Neg	150	Pos

a Sample in each role was analyzed three times, and the average value is reported in the “result” columns.

## Discussion

UDS with IA kits have a fast turnaround time to result, are easily accessible, and are typically less expensive, in comparison to mass spectrometry analysis, to identify drugs in urine and to monitor adherence to drug therapy. However, cases of false-positive UDS have been documented [12, 17–22]. Our investigation was prompted by observations of increased false-positive rates with our tapentadol UDS. The Immunalysis Tapentadol Urine EIA Drug Screening (343) Kit indicates a 0.3% cross-reactivity with tapentadol when tramadol concentrations reached 60 000 ng/mL. However, despite this reported threshold, we identified false-positive tapentadol results in 28 samples with tramadol concentrations ranging from 6901 to 297 703 ng/mL, as determined by LC–MS/MS analysis. Notably, a significant proportion (61%) of these false-positive samples exhibited tramadol concentrations below 60 000 ng/mL, suggesting a potential discrepancy between the reported cross-reactivity threshold and our observed outcomes. Interestingly, our LC–MS/MS analyses revealed that all false-positive tapentadol samples had tapentadol concentrations below cutoff, indicating that either the current tapentadol package insert claim of the no cross-reactivity with ≤60 000 ng/mL tramadol is incorrect or there are other cross-reacting contaminants This incongruity raises concerns regarding the accuracy of the previous tapentadol package insert claims and highlights the need for further elucidation of potential cross-reacting contaminants or assay limitations.

To investigate the tramadol cross-reactivity in tapentadol UDS, we conducted experiments involving spiked synthetic urine samples with tramadol. Tapentadol UDS with the reformulated New Kit-343UR showed significant improvements in its specificity over Old Kit-343, particularly in terms of reducing tramadol cross-reactivity. Our initial results indicated that Old Kit-343 produced a high rate of false positives for tapentadol in the presence of tramadol. Of note, many individuals in our test population are on tramadol than tapentadol. Although New Kit-343UR demonstrated superior performance, the false-positive tapentadol screening observed in Tap-Lo-*O*Tram-01 was not surprising based on the 100 000 ng/mL tramadol cross-reactivity mentioned in the package insert. Importantly, the retesting of 28 previously identified false-positive patient samples with the New Kit-343UR yielded no false positives, aligning perfectly with LC–MS/MS analysis results. In addition, the false-positive rate has declined since implementation of the New Kit-343UR.

Although our study clearly demonstrated tramadol-related false positives in urine tapentadol IA, determining the exact mechanism of cross-reactivity was beyond the scope of our work due to proprietary constraints on assay formulation details. Also, the specific modifications made by the manufacturer in the reformulated kit were not disclosed in the package insert. However, based on our data, we observed a dramatic reduction in tramadol-related false positives, suggesting that the changes were likely aimed at improving assay specificity. Further studies evaluating the exact nature of these modifications, if such information can be obtained from the manufacturer, would be beneficial. In addition, our interference testing was limited to tramadol and its primary phase I metabolite (*O*-desmethyltramadol). Given that glucuronidated metabolites may also contribute to cross-reactivity, additional studies assessing their impact would provide a more comprehensive understanding of tramadol interference in urine tapentadol IA. We encourage future work in this area, particularly since phase II metabolites are often abundant in urine and may have structurally relevant epitopes that contribute to antibody cross-reactivity.

This study is an example of a cautionary tale that cross-reactivity studies, conducted by the manufacturer, may not reflect the high concentrations and drug combinations that are present in authentic patient samples. It would be important for laboratories to verify manufacturer claims for cross-reactivity at higher concentrations and drug combinations reflected in the patient population. Laboratories should have a mechanism to track true and false positive rates, and it is imperative for laboratories to reach out to the manufacturer for troubleshooting if the assay does not meet expected performance. It is also important for laboratories to assess drug testing needs for clinicians to create an optimal test menu to support patient care.

## Conclusion

Our study underscores the importance of interference studies for assay verification and continuous monitoring of potential cross-reactivity in UDS assays. Further research is warranted to elucidate the underlying mechanisms of tramadol cross-reactivity and to refine tapentadol UDS methodologies, ensuring accurate and reliable detection of tapentadol in clinical practice.

## Data Availability

The data underlying this article are available in the article and in its online supplementary material.
